# Advancing Adult-Acquired Flatfoot Deformity Treatment: Enhanced Biomechanical Support Through Graphene Oxide-Integrated Bioengineered Grafts Tested In Silico

**DOI:** 10.3390/jfb15110335

**Published:** 2024-11-09

**Authors:** Sebastián Nieto, Mónica Gantiva-Díaz, María A. Hoyos, Yuliet Montoya, Juan C. Cruz, Christian Cifuentes-De la Portilla

**Affiliations:** 1 Department of Biomedical Engineering, Universidad de Los Andes, Bogotá 111711, Colombia; s.nieto4@uniandes.edu.co (S.N.); mr.gantiva@uniandes.edu.co (M.G.-D.); ma.hoyosa@uniandes.edu.co (M.A.H.); 2 Grupo de Dinámica Cardiovascular, Línea Ingeniería de Tejidos y Protésica Cardiovascular, Universidad Pontificia Bolivariana, Medellín 050031, Colombia; yulieth.montoya@upb.edu.co

**Keywords:** electrospun graft, graphene oxide, polycaprolactone, gelatin, spring ligament, finite element analysis, adult-acquired flatfoot deformity

## Abstract

Adult-Acquired Flatfoot Deformity (AAFD) is a progressive orthopedic condition causing the collapse of the foot’s medial longitudinal arch, often linked with injuries to the plantar arch’s passive stabilizers, such as the spring ligament (SL) and plantar fascia. Conventional treatment typically involves replacing the SL with synthetic material grafts, which, while providing mechanical support, lack the biological compatibility of native ligaments. In response to this shortcoming, our study developed an electrospun, twisted polymeric graft made of polycaprolactone (PCL) and type B gelatin (GT), enhanced with graphene oxide (GO), a two-dimensional nanomaterial, to bolster biomechanical attributes. The addition of GO aimed to match the native ligamentous tissue’s mechanical strength, with the PCL-GT-GO 2.0% blend demonstrating an optimal Young’s modulus of 240.75 MPa. Furthermore, the graft showcased excellent biocompatibility, evidenced by non-hemolytic reactions, suitable wettability and favorable platelet aggregation—essential features for promoting cell adhesion and proliferation. An MTT assay revealed cell viability exceeding 80% after 48 h of exposure, highlighting the potential of the graft as a regenerative scaffold for affected ligaments. Computational modeling of the human foot across various AAFD stages assessed the graft’s in situ performance, with the PCL-GT-OG 2.0% graft efficiently preventing plantar arch collapse and offering hindfoot pronator support. Our study, based on in silico simulations, suggests that this bioengineered graft holds significant promise as an alternative treatment in AAFD surgery, marking a leap forward in the integration of advanced materials science for enhanced patient care.

## 1. Introduction

The pervasive condition known as Adult-Acquired Flatfoot Deformity (AAFD) is marked by the partial or full collapse of the longitudinal medial arch of the foot [1], catalyzing progressive deformities like heel valgus and forefoot abduction [2]. These changes subsequently trigger foot pain, misalignment, and gait dysfunction [1]. Historically, AAFD has been ascribed to the deterioration of the posterior tibial tendon (PTT) [3], but a more comprehensive understanding acknowledges the role of other structures that contribute to plantar arch alignment and stability. These include the plantar fascia (PF), spring ligament (SL), talocalcaneal ligaments, and deltoid ligaments [1].

Recent studies suggest that AAFD often arises secondary to injuries of the spring ligament, either independently or in conjunction with PTT degeneration [4,5,6,7,8,9]. The spring ligament, a key ligamentous structure extending from the calcaneus to the navicular bone, serves critical functions in supporting the head of the talus, maintaining the medial arch [10], and restraining valgus deformity of the foot [3].

The Bluman–Myerson classification categorizes AAFD into four stages, each corresponding to the level of deformity and the progression of the pathology. Therapeutic interventions vary with each stage, and spring ligament injuries are predominantly associated with grade II AAFD, wherein the flatfoot remains flexible [1]. Available treatment options span from orthoses, tendon transfers, and calcaneal osteotomies to arthrodesis and spring ligament augmentation [1]. Yet certain interventions, such as midfoot fusions, may inadvertently induce secondary effects on the stiffness and flexibility of the foot, potentially exerting additional stress on the spring ligament [11]. Other types of arthrodesis may similarly exert undue strain on the forefoot and rearfoot [11], alongside the possible loss of certain biomechanical foot functions owing to tendon transfers. This underscores the pressing need to innovatively address spring ligament injuries implicated in AAFD and avert these potential complications.

Given their low cell density and restricted oxygen and nutrient supply, tendons and ligaments possess limited regenerative capacity. Hence, it is imperative to circumvent the limitations of conventional surgical techniques by developing synthetic and natural substitutes via advanced materials engineering. These substitutes ought to mimic the intricate structure and mechanical properties of native tissues while demonstrating superior biological performance [12].

Electrospinning has emerged as a promising method for fabricating polymeric nanofibers for material engineering applications, especially in ligament and tendon research. This is largely due to its capacity to replicate the fibrillar microstructure of these tissues [13], facilitating cell adhesion, proliferation, and extracellular matrix production [14,15], while enabling the amalgamation of diverse types of polymers [16]. Electrospinning is a technique used to produce nanofibers through the application of an electric charge to a polymer solution, which is then ejected as fine fibers. In this process, the polymer solution is passed through a metal needle that is connected to the positive terminal of a DC power source. Simultaneously, a target collector with a negative charge is also connected to the power source, resulting in the formation of a cone-shaped jet (referred to as the Taylor cone) that moves towards the area of lower electrical potential. As a result, the solvent evaporates from the polymer solution, leading to the deposition of nanofibers [17].

Nevertheless, one of the critical challenges associated with electrospinning is the limited mechanical strength of the resulting nanofibers, which could potentially hinder their clinical applicability [12,13,14,16,18]. To counter this, researchers have twisted or coiled electrospun nanofibers to construct more complex 3D structures with appropriate mechanical properties [12]. Moreover, the incorporation of nanocomposites such as carbon nanotubes, graphene, graphene oxide, carbon, and cellulose nanofibers [19,20] has been shown to significantly enhance the strength and longevity of the resultant structures.

**Figure 1:**
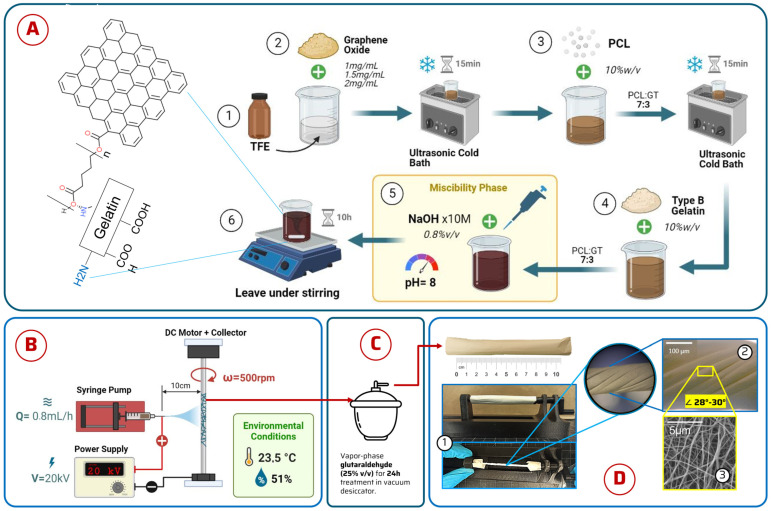
Schematic diagram of (**A**) solution preparation; (**B**) electrospinning equipment assembly and operating parameters; (**C**) vapor crosslinking method in vacuum desiccator; and (**D**) electrowinning cylindrical membrane twisting machine. (**D1**) Twisting; (**D2**) macrograph of graft; (**D3**) SEM micrograph of electrospun graft.

The goal of this study was to design electrospun grafts from biodegradable polymers as a novel strategy for reinforcing and augmenting elastic ligament injuries associated with AAFD (see Figure 1). The grafts were synthesized using a blend of synthetic and natural polymers, specifically polycaprolactone (PCL) and gelatine type B (GT), to achieve an optimal balance of strength, durability, and enhanced biocompatibility [12,16]. PCL, a synthetic polymer, offers mechanical resilience and protracted degradation time while retaining biocompatibility. Simultaneously, gelatine enhances cell adhesion, courtesy of its integrin-rich structure, which augments the biocompatibility of the graft and mitigates immunogenic responses [12]. To fortify the mechanical robustness of the graft, graphene oxide (GO), a two-dimensional nanomaterial, was incorporated. Recognized for its improved mechanical properties, increased colloidal stability, and physiologically stable, biocompatible, and antibacterial properties even at lower concentrations, GO represents an ideal component for this application [20,21]. The electrospun graft was then subjected to a battery of tests—physicochemical, morphological, mechanical, and biological—to evaluate its properties.

Furthermore, to replicate the graft’s final application, finite element analysis was undertaken to assess the insertion of the graft in a patient’s foot affected by AAFD. A computational model was formulated using CT images of a healthy patient, encompassing all foot bones, plantar fascia, cartilage, and elastic ligaments while preserving their anatomical distribution and mechanical properties. This model has been validated in several previous works, including analyzing plantar soft tissue stresses and applying different traditional surgical methods on the foot [11].

## 2. Materials and Methods

### 2.1. Materials

Polycaprolactone pellets (PCL, MW 80.000), type B gelatine (porcine skin), 2,2,2-trifluoroethanol (TFE, 99.0%), glutaraldehyde solution (grade II, 25% (*v*/*v*)), sodium hydroxide (NaOH) pellets (reactive level, >98%), phosphate-buffered saline (PBS), 3-(4,5-Dimethyl-2-thiazolyl)-2,5-diphenyl-2H-tetrazolium bromide (MTT), phosphate-buffered saline (PBS), and Triton X-100 were purchased at Sigma-Aldrich Inc. (Saint Louis, MI, USA, EE.UU). High-glucose Dulbecco’s modified Eagle medium (DMEM) was purchased from Gibco (Amarillo, TX, USA). Sodium hydroxide was purchased from PanReac AppliChem (Chicago, IL, USA). L929 P + 13 (mouse connective tissue fibroblasts) were from ATCC^®^ (Manassas, VA, USA).

### 2.2. Polymer Solution Preparation

In this study, a standardized process was utilized to prepare four distinctive polymer solutions. Initially, trifluoroethanol (TFE) served as the solvent, to which GO was added at concentrations of 1.0 mg/mL, 1.5 mg/mL, and 2.0 mg/mL. GO was synthesized from graphite oxidation/exfoliation according to the Tour Method [22]. Briefly, graphite powder was oxidized using a mixture of H_2_SO_4_/H_3_PO_4_ (9:1) with KMnO_4_, followed by the controlled addition of H_2_O_2_ and multiple washing steps with water, HCl, and ethanol. The resulting GO was characterized by FTIR and TGA to confirm successful oxidation and exfoliation. The synthesis protocol followed our previously established methods [20], yielding GO sheets with lateral dimensions in the submicron range. The mixtures underwent cold sonication for 15 min, ensuring dispersion. Then, PCL pellets were added at a concentration of 7% (*w*/*v*) and sonicated again for 15 min for homogeneity. Gelatine type B (GT) was then added at a 3% (*w*/*v*) concentration and stirred at 200 rpm for two hours. The pH of GT was adjusted to 8 using 1M NaOH to ensure miscibility. The final solution was left stirring overnight at 200 rpm [23]. A separate solution was prepared without GO for comparison. The resulting solutions were designated as PCL-GT-GO 1.0%; PCL-GT-GO 1.5%; PCL-GT-GO 2.0%; and PCL-GT for control (Figure 1A).

### 2.3. Electrospinning Setup

The electrospinning apparatus consisted of a rotating (500 rpm) cylindrical aluminum collector (20 mm diameter and 160 mm length). A total of 5 mL of the prepared solution was loaded in a syringe with a 21 G needle and pumped at a rate of 0.8 mL/h. A voltage of 20 kV was maintained across a 10 cm gap between the needle and the collector. Ambient conditions were controlled at 23.5 °C with a relative humidity of 51% during the collection process. Electrospinning proceeded until the solution was fully dispensed. Optimal electrospinning parameters were determined by iterating conditions in collector speed, voltage, and flow rate (Figure 1B).

### 2.4. Post Electrospinning Processing

Upon retrieval from the collector, the electrospun membranes exhibited a hollow cylindrical morphology. Due to GT’s aqueous solubility and the necessity for crosslinking to ensure stability at physiological temperatures, we used glutaraldehyde vapor exposure in a vacuum desiccator with 25% glutaraldehyde solution [24] for 24 h for crosslinking. For biological assays, the membranes were sterilized under UV light exposure for 40 min, cleansed overnight with 70% ethanol, and extensively rinsed with type I water to ensure suitability for subsequent assays.

### 2.5. Membrane Twisting

To improve the mechanical strength of the nanofiber membranes, we applied a textile twisting technique, manipulating the fiber architecture. The membranes, possessing a hollow cylindrical shape, were placed on a manually operated device custom-made by our 3D-printing team. The apparatus allowed for one end of the membrane to be fixed while the other was rotated to achieve a consistent torsion angle between 28° and 30° [25]. Different levels of twists were applied to create various structures: 20 twists for PCL-GT, 15 for PCL-GT-GO 1.0%, and 12 for PCL-GT-GO 2.0%.

### 2.6. Fourier Transform Infrared Spectroscopy (FTIR)

The chemical characteristics of the membranes were analyzed using Fourier Transform Infrared Spectroscopy (FTIR) on a BRUKER Alpha II A250/D system (Billerica, MA, USA). The spectra were obtained in the wavenumber range of 4000–500 cm^−1^ with a spectral resolution of 2 cm^−1^, enabling us to elucidate the chemical composition and confirm the presence of expected functional groups within the nanofibers.

### 2.7. Thermogravimetric Analysis (TGA) and Differential Scanning Calorimetry

To determine the thermal stability and decomposition profiles, the electrospun membranes were analyzed using Thermogravimetric Analysis (TGA) on an SDT Q600 (TA Instruments, New Castle, DE, USA). This analysis provided detailed information on the mass loss at various temperature thresholds from 0 °C to 650° C. Additionally, Differential Scanning Calorimetry (DSC) on a DSC Q2000 (TA Instruments, New Castle, DE, USA) was utilized to identify the thermal transitions of the materials within a temperature range of 25 °C to 400 °C. This thermal analysis helped to assess the melting and crystallization behaviors of the polymers, which are indicative of their processing stability and potential performance as graft materials.

### 2.8. Polymer Contact Angle (Wettability)

The wettability of the electrospun membranes was quantified by measuring the contact angles using a Theta Lite optical tensiometer (Biolin Scientific, Hängpilsgatan, Sweden). Employing the sessile drop method, a 3 µL water droplet was carefully placed onto each membrane’s surface. The spread of the droplet was recorded, and the contact angles were calculated with Digimizer image analysis software (v. 6.4.0)^®^ (Ostend, Belgium).

### 2.9. Morphological Analysis

The 2D morphology of the electrospun nanofibers was observed under a scanning electron microscope (SEM) (TESCAN LYRA3, Brno, Czechia) at 5 kV. Membranes prepared for SEM were subjected to ion bombardment to make their surfaces conductive. High-resolution micrographs were captured at magnifications of 5 kx, 10 kx, and 20 kx to accurately gauge the nanofiber diameters, which were then analyzed using ImageJ software (v 1.54g)^®^.

### 2.10. Hemolysis and Platelet Aggregation

The hemolysis assays were conducted following the ISO 10993-4 [26] and ASTM F 756-00 standards [26]. We prepared extracts by incubating 3 mm × 2 mm membrane samples in 2 mL of 1X PBS at 37 °C for 24 h. We drew 4 mL of blood into an EDTA tube from a healthy O+ human donor, which was then centrifuged at 1800 rpm for 5 min. After discarding the plasma and white blood cells, we replenished the tube to its original volume with 1X PBS, repeating this washing step thrice. For hemolysis testing, Triton X-100 and PBS 1X served as the positive and negative controls, representing complete (100%) and no (0%) hemolysis, respectively. We combined the extracts with the erythrocyte suspension in a 96-well plate and incubated them at 37 °C for 1 h. Post centrifugation at 3000 rpm for 5 min, the absorbance of the supernatant, indicative of hemoglobin release, was measured at 540 nm using a Multiskan FC microplate reader (ThermoFisher Scientific, Waltham, MA, USA). The percentage of hemolysis was calculated using the following formula:

$$Hemolysis\;\left(\%\right)=\frac{{Abs}_{s}-{Abs}_{\left(-\right)}}{{Abs}_{\left(+\right)}-{Abs}_{\left(-\right)}}\times 100\%$$

where Abs_s_ is the absorbance of the sample, Abs_(−)_ is the absorbance of the negative control (1X PBS), and Abs_(+)_ is the absorbance of the positive control (Triton X-100, 10% (*v*/*v*)).

Platelet aggregation was assessed by collecting 8 mL of blood from a healthy O+ donor in sodium citrate tubes and centrifuging it at 1000 rpm for 10 min to obtain plasma. Epinephrine and 1X PBS were employed as positive and negative controls to induce strong and weak aggregation, respectively. The assay involved mixing the plasma with our membrane extracts in a 96-well plate. Absorbance readings were taken using a Multiskan FC microplate reader (ThermoFisher Scientific, Waltham, MA, USA) at 610 nm. The platelet aggregation percentage was determined using the following formula:

$$Platelet\;aggregation\;\left(\%\right)=\frac{{Abs}_{s}-{Abs}_{\left(-\right)}}{{Abs}_{\left(+\right)}-{Abs}_{\left(-\right)}}\times 100\%$$

where Abs_s_ is the absorbance of the sample, Abs_(+)_ is the absorbance of the positive control (Epinephrine), and Abs_(−)_ is that of the negative control (1X PBS).

### 2.11. Cytotoxicity

To examine the cytotoxicity of the electrospun membranes, L929 P + 13 mouse connective tissue fibroblasts were cultured. A density of 1 × 10^5^ cells/mL was seeded into a 96-well microplate and incubated for 24 h in standard conditions (37 °C, 5% CO_2_) in DMEM supplemented with 5% FBS, 1% P/S. Post incubation, the cells were exposed to the various electrospun membrane samples, each providing a contact area of 12.5 mm^2^ for 24 and 48 h. DMEM served as the positive control (C+) for cellular viability, while DMSO (10% (*v*/*v*)) functioned as the negative control (C−). The MTT assay was employed to evaluate cell viability. After exposure to the MTT reagent, cells were incubated for an additional 2 h to enable formazan crystal formation (37 °C). Subsequently, these crystals were solubilized with DMSO, and absorbance was measured at 595 nm using a Multiskan FC microplate reader (ThermoFisher Scientific, Waltham, MA, USA).

The L929 mouse fibroblast cell line was selected for cytotoxicity evaluation in accordance with ISO 10993-5 [27] guidelines, which establish this cell line as a standard reference for initial biomaterial biocompatibility assessment. These cells are particularly valuable for standardized testing due to their well-characterized responses to cytotoxic substances and their documented sensitivity in detecting potential material toxicity. Furthermore, L929 fibroblasts represent a primary cellular component of connective tissues, making them particularly relevant for evaluating materials intended for ligament applications. Their use enables standardized comparison with other biomaterial studies in the literature, providing a validated foundation for biocompatibility assessment [28].

### 2.12. Finite Element Analysis Using a Foot Model

**Figure 2:**
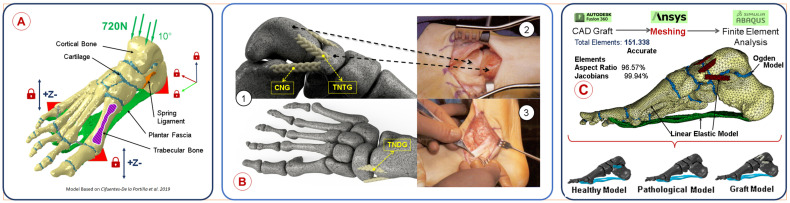
Characteristics and parameters of the computational biomechanical foot model. (**A**) Model tissues, structures, static input load, and motion constraints of the foot model [28]. (**B**) Graft placement. (**B1**) Rendering of the foot model with the 3 grafts placed in low iatrogenic surgical access structures. (**B2**) Medial midfoot incision. (**B3**) Incision with large iatrogenic effects starting at the medial malleolus along the internal axis of the foot, towards the cuneometatarsal junction [29]. (**C**) Meshing characteristics of the foot model, implemented models, and software used for graft design. Healthy model: Foot model with SL and PF native properties. Pathological model: Foot model without SL. Graft model: Foot model without SL and hindfoot grafts.

To evaluate the use of the graft designed, we performed some simulations using a computational model of the human foot previously used and validated by our research group to study AAFD progression. The model reproduces the foot of a 49-year-old male subject, weighing 720 N and standing 170 cm tall, preserving the natural morphology of the cartilage, cortical and trabecular bone, plantar fascia, and spring ligament [28] (see Figure 2A). The model has been validated for both healthy and pathological conditions. For this study, we positioned graft geometries in various foot regions, aiming for more dorsal locations while preserving the spring ligament’s insertion area. We made graft placement decisions based on three criteria: a. the grafts should replace the spring ligament’s passive function in preventing medial arch drop; b. the grafts should perform the passive work of the LS in hindfoot pronator stability; and c. the grafts should be located in areas that prevent iatrogenic effects during access and surgical anchoring to bone structures.

We modeled three types of grafts, designed in Fusion360 (Autodesk, Mill Valley, CA, USA), with a double helical morphology to mirror the macroscopic structure of the electrospun grafts post-torsional treatment. The graft placement was guided by the advice of expert foot and ankle surgeons and was based on medical images of surgical procedures from Techniques in Orthopaedic Surgery [29]. The grafts were named and positioned as follows: Calcaneonavicular Graft (CNG): Located between the sustentaculum tail of the medial aspect of the calcaneus and the navicular tuberosity. This graft is closest to the LS’s native location (See Figure 2(B1)). Talonavicular Tuberosity Graft (TNTG): Positioned between the navicular tuberosity and the talus. This graft prevents sagging of the medial longitudinal arch and supports the surrounding grafts (See Figure 2(B1)). Talonavicular Dorsal Graft (TNDG): Located in the foot’s most dorsal area, between the navicular and the talus. This graft works in tandem with the other grafts to prevent rearfoot pronation and the fall of the medial longitudinal arch (See Figure 2(B1)).

The mesh of the grafts models was generated using linear tetrahedral elements (C3D4) with a maximum size of 0.5 mm in Ansys (Ansys V.2121.R1, Canonsburg, PA, USA) (see Figure 2C). We checked the mesh quality according to the guidelines described by Burkhart [30].

The mechanical characteristics of the tissues included on the computational model are described in Table 1. We modeled the bone (cortical and trabecular), spring ligament, and plantar fascia as elastic–linear (E-L), whereas we considered the cartilage to be nonlinear and hyperelastic [11].

The Ogden model assigned to the cartilage material considers the strain energy density function U, described by following equation:

$$U=\frac{\mu }{\alpha^{2}}\left(\lambda_{1}^{\alpha }+\lambda_{2}^{\alpha }+\lambda_{3}^{\alpha }\right)+\frac{1}{D}{\left(J-1\right)}^{2}$$

where µ is the initial shear modulus, α is the strain hardening exponent, and D is the compressibility parameter. The values used are µ = 4.4, α = 2, and D = 0.45. The model works by applying a load of 720 N, representing the total weight of an adult person in monopodal support. This load is distributed in the tibio-ankle (90%) and peroneal–ankle (10%) contact zone. The constraints for our model were established by locking all the degrees of displacement at the base of the calcaneus and by inhibiting the vertical displacement (Z axis) of the first and fifth metatarsal bases [28].

We carried out the simulation and biomechanical analysis of the computational model using Abaqus (Dassault Sistemes, Velizy-Villacoublay, France). The parameters for evaluation were selected in two directions: a. biomechanical tissue stress: stress on the plantar fascia, and b. structural foot displacements: medial longitudinal arch fall and fore/hind foot displacements (foot abduction/adduction performance).

These were considered across three distinct models: a healthy model (preserves all foot structures and healthy mechanical properties of the native tissues, including the LS), a diseased model (mimics an injured LS scenario, does not consider the work carried out by the LS, other structures maintain their mechanical properties), and the grafted model (an iterative model of the three grafts, disregards the LS, other structures maintain their mechanical properties) (Figure 2C).

We assessed the biomechanical tissue stress under the principal maxima criterion; thus, the tensile loads of the plantar fascia were calculated to identify the contribution of the LS and grafts in the pathological scenario. We measured the structural deformation of the foot at the sagging of the longitudinal medial arch by placing a sensor-node on the medial head of the talus and measuring the vertical displacement (Z axis).

### 2.13. Statistical Analysis

Statistical analysis was performed using GraphPad Prism 8^®^ (GraphPad Software, San Diego, CA, USA). The data, particularly related to the impact of varying graphene oxide concentrations on the properties of electrospun membranes, were presented as mean values with their corresponding standard deviations. One-way ANOVA followed by Tukey’s multiple comparison tests were utilized to discern the statistical significance between the groups. This analytical approach ensures a rigorous evaluation of the treatment effects, validating the robustness of the experimental outcomes.

## 3. Results

### 3.1. Synthesis of Electrospun Grafts

**Figure 3:**
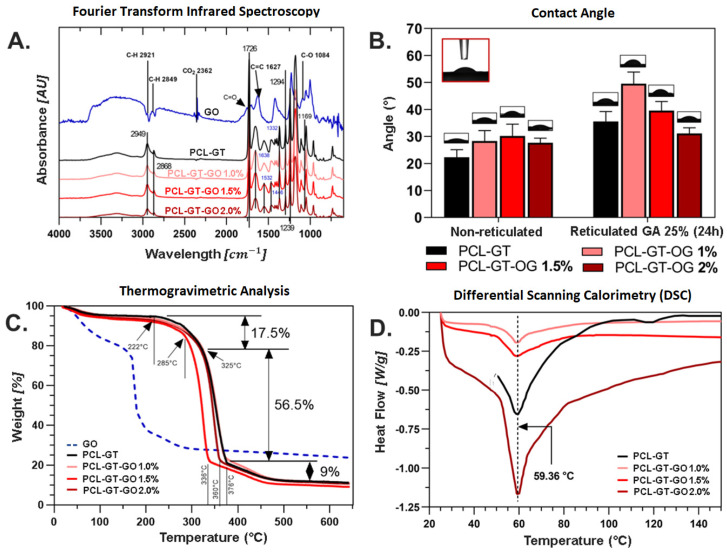
(**A**) Absorbance FTIR spectra for graphene oxide and electrospun grafts in each of their treatments and (**B**) evaluation of wettability in all electrospun membranes by means of contact angle test with deionized water and optical images associated with each graft. (**C**) Thermal analysis results TGA (0–650 °C). (**D**) Thermal analysis results DSC (25–400 °C) for all electrospun grafts.

The integration of GO into electrospun scaffolds was effectively validated using Fourier Transform Infrared (FTIR) Spectroscopy and thermogravimetric analysis (TGA). The FTIR spectra, displayed in Figure 3A, reveal distinct absorption bands for various PCL-GT-GO formulations alongside a GO control. Hydroxyl group stretching vibrations are evident in the 3000–3500 cm^−1^ range, signifying the presence of GO. Aromatic peaks at 1627 cm^−1^, attributed to C=C bond stretching, along with bands at 1726 cm^−1^ and 1084 cm^−1^ for C=O and C-O bonds, respectively, confirm the synthesis process’s efficacy in retaining oxygen-rich functional groups. Amide bonds from gelatin incorporation are discernible at 1640 cm^−1^ (amide I) and 1540 cm^−1^ (amide II), corroborating successful polymer integration [2]. The spectrum also includes peaks that suggest PCL’s presence, such as at 1740 cm^−1^ for the carbonyl group’s stretching vibration and 2875 cm^−1^ and 2940 cm^−1^ for CH_2_ bond vibrations, along with other PCL-related bands at 1230 cm^−1^ (asymmetric stress vibration of the C-O-C bond) and 1300 cm^−1^ (stretching vibration of the C-O and C-C bonds) [2,32,33,34].

The TGA results, depicted in Figure 3C, indicate a prominent weight loss at around 325 °C across all grafts, ascribed to PCL’s ester chain cleavage, yielding H_2_O, CO_2_, and 5-hexenoic acid. A secondary weight reduction, approximately 9%, concludes around 450–460 °C, linked to ε-caprolactone production from depolymerization [35]. Incorporating GO alters this behavior; GO-containing grafts undergo earlier mass loss due to GO decomposition at about 163 °C, where carboxyl groups break down to release CO_2_ [36]. DSC analyses show a consistent melting temperature near 59.36 °C for all grafts, suggesting GO’s addition does not significantly alter the thermal properties of the electrospun material (Figure 3D).

### 3.2. Polymer Contact Angle (Wettability)

The wettability of the electrospun graft surfaces was evaluated by measuring contact angles, as depicted in Figure 3B. Contact angles less than 90° typically denote hydrophilic materials, while angles greater than 90° suggest hydrophobic properties. Following glutaraldehyde vapor crosslinking treatment, a marked decrease in hydrophilicity was noted among the grafts, likely as a result of chemical crosslinking within gelatin’s polymeric chains. However, it is noteworthy that none of the graft samples exhibited contact angles exceeding the 90° threshold.

In samples containing GO, the post-crosslinking increase in contact angle was mitigated. For example, the contact angle of the PCL-GT graft diminished from 35.6° to 31.1° upon the addition of 2% GO (PCL-GT-GO 2%). This suggests that GO contributes to preserving the wettability of the grafts even after undergoing chemical crosslinking.

### 3.3. Mechanical Evaluation

Mechanical characterization of our electrospun grafts indicated compaction within the fibers corresponding to the helical orientation of polymer chains under mechanical stress. In samples with increased GO concentration, we observed a reduction in ultimate stress but an increase in stiffness. The mechanical properties were evaluated at two critical stages of graft preparation: the initial electrospun mat and the final twisted yarn configuration. The electrospun mat in its untwisted form (length = 12.38 mm; height = 5.7 mm; thickness = 0.28 mm) showed average tensile strengths of 1.375 MPa, with Young’s moduli between 6.8 and 40.5 MPa across different GO concentrations. Following the twisting process, which was designed to better mimic the hierarchical structure of native ligament tissue, the mechanical properties were significantly enhanced. The twisted yarn configuration exhibited increased tensile strengths (ranging from 7.7 to 16.8 MPa) and higher Young’s moduli (125 to 275 MPa), with the PCL-GT-GO 2.0% formulation achieving the optimal value of 240.75 MPa, closely aligning with the mechanical properties of native tissues among all tested samples. These properties made this sample a prime candidate for grafting model simulations aimed at flatfoot pathology applications. This enhancement can be attributed to the compaction and alignment of fibers during the twisting process, leading to more efficient load distribution throughout the structure. The dramatic improvement in mechanical properties after twisting underscores the importance of this processing step in achieving properties suitable for ligament applications.

**Figure 4:**
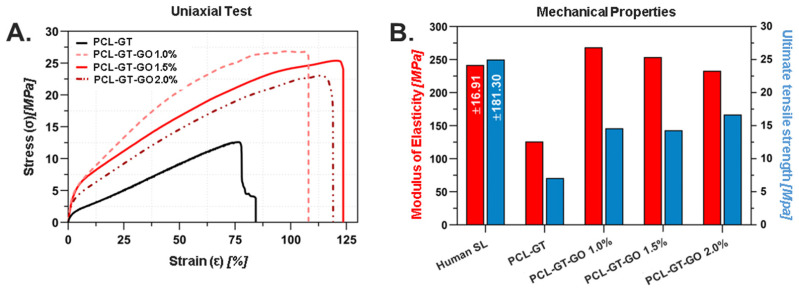
Characterization of the mechanical properties of electrospun grafts. (**A**) Stress–strain curves in a tensile test. (**B**) Elastic modulus and tensile stress at break.

Figure 4A illustrates the mechanical properties of the grafts, including the modulus of elasticity and ultimate stress at break. The inclusion of GO across all grafts resulted in enhanced mechanical performance relative to samples comprising solely PCL and GT. The most significant improvement was observed in the PCL-GT-GO 1% graft, which displayed an ultimate tensile strength increase of 112% over the PCL-GT control.

Our findings suggest that the methodical integration of GO within the polymeric matrix and the strategic fiber twisting process contribute substantially to the observed mechanical enhancements. This improvement is crucial for the potential use of these grafts as temporary reinforcement structures and as scaffolds for tissue regeneration in ligament injuries.

### 3.4. Biocompatibility

**Figure 5:**
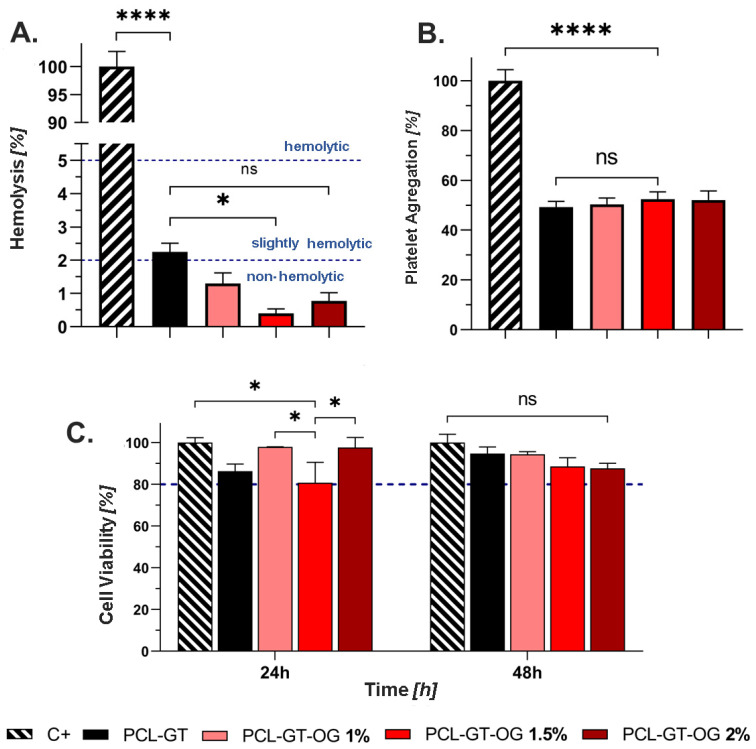
Biocompatibility results: (**A**) Hemolytic behavior, (**B**) platelet aggregation, and (**C**) cell viability for PCL-GT, PCL-GT-GO 1%, PCL-GT-GO 1.5%, and PCL-GT-GO 2%. The symbol * corresponds to a statistically significant difference with a *p*-value in the range of 0.01 ≤ *p*-value ≤ 0.05, **** to *p*-value < 0.0001.

In assessing the biocompatibility of electrospun grafts, hemolytic behavior and cytotoxicity were crucial parameters. The hemolysis tests, adhering to ASTM F 756-00 standards, revealed that while PCL-GT grafts were slightly hemolytic, grafts with any concentration of GO were deemed non-hemolytic. Specifically, the PCL-GT-GO 1.5% variant demonstrated a notable reduction in hemolytic activity compared to PCL-GT grafts, suggesting improved compatibility with blood (Figure 5A) [37].

Platelet aggregation assays indicated that all grafts, regardless of GO concentration, function as medium aggregators, with no substantial differences in aggregation behavior across different GO treatments. Such a medium level of aggregation is beneficial for supporting tissue healing processes (Figure 5B) [38].

Cell viability assays utilizing the L929 P + 13 mouse fibroblast cell line showed promising results. Within the first 24 h, all grafts maintained over 80% viability. There were significant viability differences noted between the control group and the PCL-GT-GO grafts, especially the 1.5% concentration, indicating a possible concentration-dependent effect of GO on cell viability. By 48 h, cell viability had improved across all samples, with no significant differences noted among them, indicating good cytocompatibility (Figure 5C).

### 3.5. Morphological Analysis

Scanning electron microscopy revealed the surface morphology of the electrospun grafts, showcasing stable nanofibers with minimal defects and a largely random orientation that mimics the natural architecture of connective tissue [39]. The measured average fiber diameters ranged from 161 nm to 115 nm across the different graft formulations. Specifically, the diameters for PCL-GT, PCL-GT-GO 1%, PCL-GT-GO 1.5%, and PCL-GT-GO 2% specimens were quantified, with the smallest diameter observed in grafts with the highest GO concentration.

Notably, a trend of decreasing fiber diameter with increasing GO content was observed, except for the PCL-GT-GO 2.0% sample which, counterintuitively, showed larger fiber diameters than the grafts with lower GO concentrations [40]. This unexpected result was consistently replicated across multiple observations and measurements, indicating a potential variation in the dispersion of GO within the samples.

**Figure 6:**
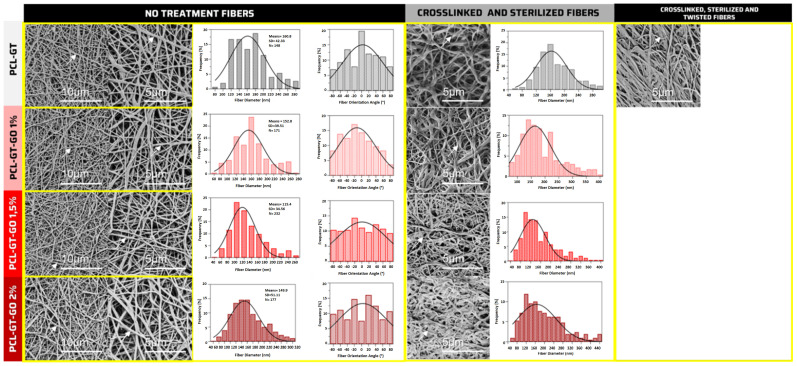
Measurement of nanofiber diameters after crosslinking from the surface morphology of electrospun membranes using SEM micrographs. From top to bottom, the order of grafts is PCL-GT, PCL-GT-GO 1.0%, PCL-GT-GO 1.5%, and PCL-GT-GO 2.0%. From left to right, the images show no treatment fibers (fiber diameter and fiber orientation angle), crosslinked and sterilized fibers, and crosslinked (fiber diameter), sterilized, and mechanically twisted fiber treatment (fiber orientation angle). Insert plots: histograms and Gaussian fit of fiber diameters.

Figure 6 presents a series of micrographs that visually document these findings, with the stable fiber formation and uniformity clearly displayed. These images are integral for validating the consistency of the electrospinning process and ensuring the reproducibility of the scaffold’s nanofibrous structure.

### 3.6. Finite Element Analysis

**Figure 7:**
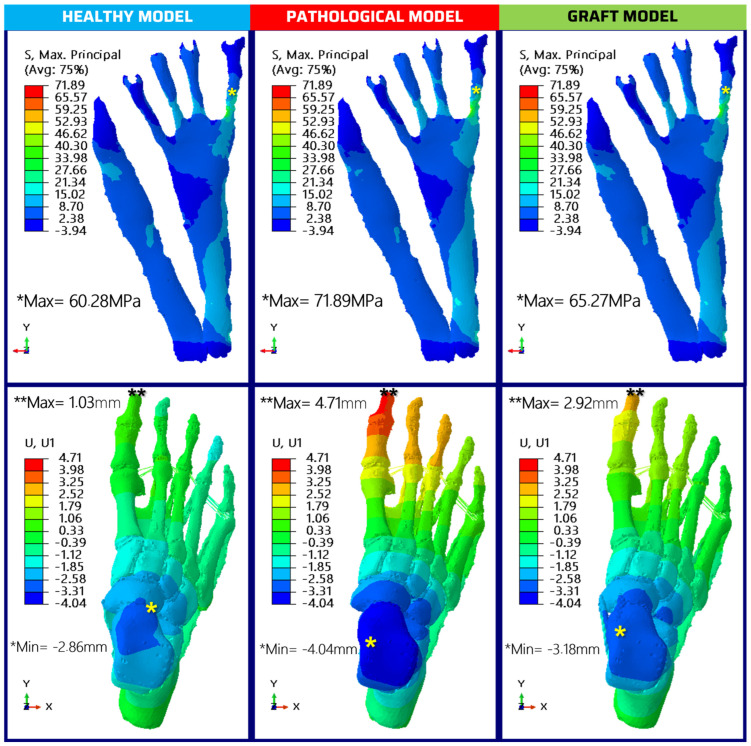
Top: Results of the evaluation of principal maxima in the plantar fascia for the three models. Bottom: Analysis of foot displacements in the X axis (evaluation of lateral displacements in the rearfoot and forefoot) for the three models. Asterisks mark the location of the maxima (**) and minima (*).

Adult-Acquired Flatfoot Deformity is typically marked by the progressive weakening and subsequent collapse of the foot’s medial longitudinal arch [40]. The condition is complex and often impairs the individual’s gait and balance. Through computational modeling, the vertical displacement of the talus—a critical indicator of arch integrity—was meticulously evaluated. Results can be seen in Figure 7.

In the healthy foot model, the talus node experienced a displacement of −4.32 mm. By contrast, the model representing the diseased state exhibited a more pronounced collapse, with a 10% increase in displacement, measuring −4.75 mm. This metric clearly illustrates the mechanical compromise associated with AAFD. Yet when the model was adjusted to incorporate the proposed grafts, there was a notable correction in the pathological drop, with a reduction to −4.44 mm, indicating a 6% improvement.

It is essential to consider the interconnected nature of the foot’s arches; the failure of one arch can precipitate a cascade of biomechanical dysfunctions throughout the structure [1]. Despite this, the lateral longitudinal arch’s collapse, another potential area of concern, did not significantly differ across the analyzed models (See Table 2). This finding could indicate that the corrective measures primarily influence the medial arch, leaving the lateral arch relatively unaffected, or that the model’s scope did not extend to detectable changes in the lateral arch. This nuanced understanding of AAFD progression and the correction potential of grafts offers valuable insights for clinical interventions aimed at restoring foot stability and function.

## 4. Discussion

In the quest to address AAFD, a complex condition characterized by the breakdown of the foot arch’s structural integrity, this study embarks on an innovative approach. Traditional interventions, such as the reinforcement or replacement of the spring ligament, often utilize synthetic material grafts for mechanical support, yet these lack the biological affinity inherent to native tissues. To bridge this gap, our research has homed in on a novel graft fabrication methodology. We have engineered a composite, twisted polymeric graft that integrates PCL and type B gelatin (GT) with GO, a reinforcement agent, aimed at amplifying the graft’s biomechanical properties.

This graft is meticulously designed to emulate the biomechanical support furnished by the natural spring ligament, which plays a pivotal role in maintaining the arch structure. The inclusion of GO not only enhances the mechanical strength of the graft but also contributes to its overall stability and longevity—traits imperative for an effective treatment modality for AAFD. Through this synergy of PCL’s robustness, GT’s biocompatibility, and GO’s structural enhancement, we present a promising therapeutic alternative that may transcend the limitations of current synthetic grafts. The results showed that the incorporation of GO into polymeric scaffolds enhances both mechanical and biological properties, which is pivotal for their successful application in tissue engineering and regenerative medicine, particularly for surgical interventions such as those for AAFD. This study’s electrospun grafts, augmented with GO, displayed improved mechanical strength and a commendable biological compatibility profile. The synthesis process preserved the intricate structures of both the polymeric matrix and the embedded GO, as substantiated by the characteristic absorption bands and peaks in the FTIR spectra, which are in line with the oxygen-rich functional groups’ resonances resulting from the synthesis of GO [41]. The sustained mechanical integrity of the grafts after GO integration is of particular relevance given that the mechanical support is critical for replacing ligaments in AAFD.

Furthermore, the TGA data confirm that our electrospun materials exhibit appropriate thermal degradation points, similar to native ligamentous tissue, suggesting that the structural integrity of the grafts would be maintained under physiological conditions [42]. The consistent melting temperatures reported in the DSC curves reaffirm that the integration of GO into the scaffolds does not adversely affect their thermal stability, echoing findings from other works that have explored the incorporation of GO into various polymeric matrices for biomedical applications [41]. Together, these results affirm the potential of these GO-enhanced electrospun grafts to serve as suitable candidates for ligament replacement in AAFD surgeries. Their physicochemical attributes promise a graft that can withstand biomechanical forces while fostering favorable cellular interactions, a necessary condition for the long-term success of implantable devices [43].

The observed wettability of the electrospun grafts in this study is of paramount importance, as it influences the material’s interaction with biological fluids and cells. Contact angle measurements yielded results that highlight the hydrophilic nature of our grafts, even after glutaraldehyde vapor crosslinking, with angles consistently below 90°. This hydrophilicity is crucial for biomedical applications, particularly those involving direct contact with cells and tissue, as it promotes better adhesion and proliferation of cells. The chemical crosslinking of gelatin’s polymeric chains seems to induce a decrease in hydrophilicity, which is counteracted by the inclusion of GO. Notably, the PCL-GT-GO 2% demonstrated a significant retention of hydrophilicity post crosslinking, which can be attributed to the nanoscale roughness and high density of oxygen-containing functional groups on GO. This result aligns with previous studies indicating that GO’s unique structure and chemistry can influence surface wettability and enhance biomolecule adsorption [44,45].

Furthermore, the addition of GO to the grafts likely contributes to the biomaterial’s bioactivity. GO is known for its ability to adsorb biomolecules, owing to its functional groups, which provide binding sites for proteins and other cellular components. This interaction is not just a passive physical adsorption but can lead to bioactive conformational changes in the adsorbed biomolecules, thus impacting cellular responses [46,47,48]. In the context of muscle cell adhesion, GO’s surface properties are hypothesized to enhance muscle cell binding capacity, potentially through the adsorption of extracellular matrix proteins that mediate cell–material interactions. Such properties are invaluable in tissue engineering applications where promoting cell–material interactions is pivotal for the integration and functionality of the implanted material [49].

The implications of these findings are twofold. Firstly, the maintenance of hydrophilicity and bioactivity in graft materials post crosslinking is instrumental for their use in vivo, where they must interact favorably with the host environment. Secondly, the nuanced understanding of how GO modifies these properties can inform the design of future biomaterials that leverage these interactions to enhance cell adhesion and proliferation, leading to improved outcomes in tissue engineering and regenerative medicine.

As the GO concentration within a polymer matrix increases, our findings reveal that ultimate stress tends to decrease, whereas stiffness, a critical factor for robust biomaterials, augments. Such an increase in stiffness was observed in the PCL-GT-GO 2% sample, which boasted a stiffness value of 240.75 MPa, closely resembling the mechanical characteristics of native ligamentous tissue. This underscores the potential of GO-reinforced polymers in tissue engineering, particularly for applications demanding high-strength materials such as ligament scaffolds for AAFD. The improved mechanical properties, including the modulus of elasticity and the ultimate stress at break observed in the PCL-GT-GO 1% graft, indicate a 112% enhancement over the PCL-GT sample alone [50]. This substantial improvement is largely attributed to the homogeneous dispersion of GO within the fibers, which is a critical aspect in the reinforcement of polymers [51]. Furthermore, the twisting process applied to the fibers plays an integral role in achieving the desired stiffness properties, making the developed graft a viable temporary reinforcement solution as well as a scaffold for tissue regeneration.

The development of biocompatible grafts, particularly for integration with human tissues, is complex and requires balancing mechanical attributes with biological compatibility. The current study’s findings indicate that incorporating GO into electrospun grafts substantially affects these aspects, promoting an environment suitable for tissue regeneration. For hemocompatibility assessment, the PCL-GT-GO 1.5% grafts showed significantly reduced hemolytic behavior compared to PCL-GT, aligning with the standards of ASTM F 756-00. This reduction in hemolysis is crucial as the rupture of erythrocytes and the resulting release of hemoglobin can have adverse clinical effects. The role of GO in reducing hemolytic activity has been affirmed by its success in various biomedical applications, as highlighted in a study on graphene oxide-polymer aerogels for their hemostatic potential [52]. Additionally, blood-compatible properties of GO have been demonstrated in a superhydrophobic graphene/titanium dioxide coating study, emphasizing its non-cytotoxicity and compatibility with blood-contacting applications [53].

In terms of platelet aggregation, the medium aggregation profile of our grafts is of particular importance, as this is crucial for healing. GO’s introduction did not significantly alter the aggregation behavior, ensuring that the grafts support chemotactic gradients essential for the migration and differentiation of stem cells. Such aggregation is optimal for stem cell migration and differentiation, as it helps in tissue regeneration—a vital aspect emphasized by the work on graphene oxide’s impact on anticoagulation and the hemolytic properties of human blood [54].

The L929 P + 13 mouse fibroblast cell line was utilized for cytotoxic assays due to fibroblasts being the main cellular component of dense connective tissues [55]. Cell viability above 80% across all grafts after 48 h indicates the favorable interaction of cells with the material, a result that aligns with other studies which discuss graphene-based materials as keys for successful application in blood-contacting devices [38]. The selection of a 48 h timeframe for cell viability assessment follows ISO 10993-5 guidelines and captures the critical initial period of cell–material interaction. During this period, cells are particularly susceptible to any cytotoxic effects from material degradation products or leachable components [56]. The maintained cell viability above 80% throughout this period, coupled with our material stability data from thermal and mechanical analyses, suggests favorable long-term biocompatibility prospects. While extended in vitro and subsequent in vivo studies would provide additional insights into sustained biocompatibility, our current results establish a strong foundation for the material’s biological safety. This is particularly relevant given that the initial 48 h represent a crucial window for cell survival and attachment, which are prerequisites for successful long-term tissue integration.

Regarding the long-term biological implications of GO in our composite, several factors contribute to its expected safety profile. The GO in our system is firmly integrated within the PCL-GT polymer matrix, rather than existing as free nanoparticles, which significantly reduces potential systemic distribution. Recent studies have shown that matrix-bound GO primarily remains localized at the implantation site, with minimal risk of particle dissemination [38]. Furthermore, the gradual degradation of PCL allows for controlled exposure of GO components, while the presence of cellular machinery capable of biodegrading graphene-based materials through peroxidase-mediated mechanisms has been documented [49]. The concentration of GO used in our system (maximum 2.0%) is well within the range demonstrated to be safe in long-term implantation studies. Additionally, our biocompatibility results showing negligible hemolytic activity and sustained cell viability support the material’s biological safety. Nevertheless, we acknowledge that comprehensive long-term in vivo studies would provide valuable additional information about the material’s degradation patterns and potential systemic effects.

Overall, the addition of GO contributes not only to the mechanical robustness of the grafts but also enhances their biological performance [57]. The ability of GO to maintain hydrophilicity post chemical crosslinking is a notable finding, reinforcing the idea that GO can facilitate biomolecule retention and cell-binding capacity—this adaptability of GO is instrumental in encouraging favorable cellular behavior. The potential for GO-enhanced electrospun grafts in clinical applications for ligament repair and tissue engineering has been further validated through in vivo studies on the toxicity and blood compatibility of GO nanoparticles [57].

The morphological intricacies of electrospun grafts are pivotal for their biological and mechanical performance. Our analysis revealed stable, defect-minimized nanofibers that effectively mimic the dimensions of natural connective tissue fibrils. A notable reduction in nanofiber diameter was observed with increasing GO concentrations. This reduction aligns with findings on the effects of graphene oxide on electrical conductivity and attraction forces during electrospinning, which are essential for creating biomimetic structures [58].

However, disparities in fiber size within the PCL-GT-GO 2.0% sample suggest a non-uniform dispersion of GO, a phenomenon that has been investigated for its impact on the uniformity of nanofiber properties [59]. The implications of this non-uniformity are significant, as uniform dispersion is crucial for achieving consistent performance, especially in biomedical applications where scaffold architecture can affect cell adhesion and growth.

The optimal nanofiber diameter for wound dressing materials, as highlighted by [60], emphasizes the importance of these findings. Moreover, the challenge of achieving a balanced fiber morphology is discussed by [61], who demonstrate the complex relationship between electrospinning parameters and the resultant nanofiber structure.

The mechanical behavior of foot structures under stress, particularly the plantar fascia (PF), is crucial for understanding foot dynamics and pathologies. The PF’s role as a primary passive stabilizer of the plantar arch makes it a focal point in biomechanical studies and simulations. In this context, our study’s computational model, as illustrated in Figure 7, quantified the tensile stress in the PF, revealing a baseline stress of 60.28 MPa against a pathological model. This metric is in line with observations from previous studies, such as those conducted by McDonald et al. (2016), where the significance of PF stress in running mechanics was investigated, indicating similar baselines for healthy tissue [62].

The diseased state, characterized by a compromised spring ligament, showed an excess stress of 19% (11.61 MPa) transferred to the PF, highlighting the ligament’s integral support role. This corroborates the findings presented by Pontious et al. (1996), which emphasized the critical stabilizing function of the spring ligament and its influence on adjacent structures [63]. Incorporating the proposed graft in our model resulted in an 11% reduction in excess stress on the PF, thereby validating the graft’s mechanical effectiveness. This mirrors the biomechanical implications discussed in research presented by Gu et al. (2013), where the role of the PF in distributing plantar stress was explored, reinforcing the benefits of targeted interventions [64].

Lateral deformation assessments provided insights into the foot’s pronation and abduction patterns. In healthy models, the left lateral displacement (*Min) at the talus-navicular joint depicted the pronatory movements of the hindfoot. Displacement shifts in diseased models toward the medial region highlighted the altered biomechanical lever arm, reflecting findings by Beltran (2022), which discussed the implications of medial shifts on foot stability [8,65]. Maximum right lateral displacements (**Max) observed in distal phalanges indicated abduction motions. The grafted model’s correction of this displacement by 112% indicated a restorative effect, closely aligning with simulations performed by McDonald et al. (2016), demonstrating the corrective capabilities of biomechanical interventions on foot deformation. Collectively, these results not only showcase the mechanical resilience of the graft in simulating healthy foot mechanics but also its potential as a clinically viable option for AAFD management in its early stages. Such potential is corroborated by recent advancements in biomaterials and tissue engineering that emphasize the importance of mimicking natural biomechanics in synthetic grafts, as discussed previously [15,62].

While our mechanical characterization focused primarily on tensile properties, which represent the predominant physiological loads experienced by the spring ligament, we acknowledge that fatigue and creep behavior are also relevant for long-term ligament applications. The recent literature on graphene oxide-reinforced composites suggests that materials demonstrating enhanced tensile properties, particularly those achieving native tissue-like elastic moduli, typically exhibit favorable fatigue resistance and creep behavior [50,51]. The achievement of a Young’s modulus of 240.75 MPa in our PCL-GT-GO 2.0% formulation, matching native ligament tissue properties, suggests promising mechanical durability. This is further supported by our computational modeling results, which demonstrate the graft’s ability to withstand physiological loads while maintaining structural integrity.

As shown in Table 3, our developed PCL-GT-GO graft offers several advantages compared to current clinical solutions for AAFD treatment. While traditional tendon transfers provide excellent biocompatibility, they are limited by donor site morbidity and availability. Synthetic grafts, though offering high initial strength, often lack optimal biological integration. Allografts present immunological concerns and availability limitations. Our PCL-GT-GO graft addresses these limitations by providing mechanical properties matching native tissue (240.75 MPa), excellent biocompatibility (>80% cell viability), and controlled degradation properties, without the constraints of donor site morbidity or tissue availability. Furthermore, the customizable nature of our graft’s properties through GO concentration adjustment offers potential advantages for patient-specific treatment approaches.

The degradation profile of our composite graft is expected to be advantageous for ligament tissue engineering applications. PCL, our primary polymer component, exhibits a degradation timeline of 18–24 months through hydrolytic degradation of ester linkages [16], which aligns well with typical ligament healing and remodeling timeframes. The incorporation of GO has been shown to modulate polymer degradation rates, with studies indicating that GO can enhance the hydrolytic stability of polymer matrices while maintaining biocompatibility [49]. Additionally, the presence of gelatin in our composite introduces enzymatically degradable components, potentially creating a more biomimetic degradation profile. This combination of materials is designed to provide sustained mechanical support during the critical healing period while allowing for gradual tissue ingrowth and matrix remodeling. While specific degradation studies would provide valuable additional information, the established degradation characteristics of our constituent materials suggest a favorable profile for ligament tissue engineering applications.

One of the limitations of this study is that we did not include experimental data about the presence of GO as raw material once in the electrospun material. However, we have provided comprehensive characterization of GO integration through complementary analytical techniques, building upon our established expertise in GO characterization as demonstrated in our previous works [20,66]. It is important to acknowledge that while our study provides comprehensive in vitro characterization and computational modeling of the graft’s performance, future in vivo investigations would offer valuable additional insights into its behavior in a physiological environment. Our current approach combining in vitro testing and computational modeling represents an essential first step in the development pipeline, aligning with established protocols in biomaterial development. The robust mechanical, biocompatibility, and simulation data presented here establish a strong foundation for subsequent in vivo studies, which could further validate the graft’s potential for clinical applications. The promising results from our hemolysis, platelet aggregation, and cell viability assays, coupled with the favorable mechanical properties and computational predictions, suggest that the graft warrants further investigation in physiological conditions.

## 5. Conclusions

Our study presents a novel GO integrated polymeric graft, revealing quantitatively superior mechanical and biological properties compared to traditional PCL-GT scaffolds. The PCL-GT-GO 2% formulation exhibited a significant stiffness value of 240.75 MPa, mirroring the mechanical robustness of native spring ligaments and suggesting potential applicability in reinforcing larger ligaments, such as the ACL, which are subject to substantial mechanical loads. For ligaments bearing less mechanical stress, like those in grade I or II injuries, our grafts not only provide temporary structural support but also show promising results as scaffolds for cellular growth and tissue regeneration. The biocompatibility of these grafts is underscored by negligible hemolytic activity (hemolysis < 2%) and favorable platelet aggregation, which are critical for facilitating the natural healing process through chemotactic signaling and cellular migration. Furthermore, the grafts maintained high cell viability, with more than 80% of cells remaining viable after 48 h in an MTT assay. This is largely attributed to the hydrophilic nature of the natural polymer GT in the graft composition, which significantly enhances cell adhesion—a finding confirmed by wettability measurements, where the PCL-GT-OG 2% formulation demonstrated a contact angle conducive to cellular interaction (31.1°). Biomechanical simulations provided quantitative evidence of the grafts’ efficacy in situ. Within the mechanically demanding environment of the medial longitudinal arch, the PCL-GT-OG 2% graft commendably mimicked the biomechanical behavior of native structures, with only an 11% deviation in talus displacement compared to the healthy model.

In the larger context of ligament repair and tissue engineering, our findings suggest that PCL-GT-GO grafts have a strong potential as both temporary reinforcement for acute ligament injuries and as a scaffold for sustained tissue regeneration. Their mechanical resilience combined with exceptional biocompatibility and favorable cell interaction characteristics could herald a new era in the treatment of ligament-related pathologies. Our study advocates for further research to explore the full potential of these advanced biomaterial grafts across a spectrum of biomechanical applications. This work provides a compelling proof-of-concept that could pave the way for the development of next-generation ligament repair strategies, potentially improving outcomes for patients worldwide.

## Figures and Tables

**Table 1:** Mechanical properties of the elastic–linear tissues of the human foot model. * PCL-GT-OG 2.0%. Values from [28,31].

Tissue	Elasticity Modulus	Poisson Ratio
Cortical bone	17,000 MPa	0.3
Trabecular bone	700 MPa	0.3
Spring ligament	250 MPa	0.28
Plantar fascia	240 MPa	0.28
Grafts *	240.7 MPa	0.3

**Table 2:** Results of the foot model simulations, using the healthy model (SL + PF), pathological model (PF only), and all iterations in all combinations of the three grafts. The results of the healthy model are given in physical magnitudes; all other values are represented in percentages (%). These percentages represent the differential increase compared to the healthy model (SL + PF).

Foot Model	Medial Long Arch Fall (mm)	Foot Displacement (mm)		Plantar Fascia Stress (MPa)
	Displacement	Forefoot	Hindfoot	Max. Principal
SL + PF (healthy)	4.32	1.03	2.86	60.28
PF only (pathological)	9.80	357.78	41.20	19.26
CNG + PF	5.49	183.95	19.80	10.57
TNTG + PF	9.49	343.48	39.98	18.64
TNDG + PF	7.14	368.29	32.46	17.40
CNG + TNTG + PF	5.36	180.74	19.03	10.36
CNG + TNDG + PF	2.78	192.80	11.33	8.56
TNTG + TNDG + PF	6.87	347.08	31.76	16.63
CNG + TNTG + TNDG + PF	2.68	184.44	11.12	8.27

**Table 3:** Comparison of current clinical solutions with the developed PCL-GT-GO graft for AAFD treatment.

Treatment Approach	Mechanical Properties	Biological Response	Clinical Considerations
Tendon Transfer [1]	Native tissue properties (150–300 MPa)	Excellent biocompatibility	Risk of donor site morbidity; Limited availability
Synthetic Grafts (e.g., LARS) [51]	High initial strength (>400 MPa)	Limited cell adhesion; Risk of foreign body response	No donor site morbidity; Risk of long-term failure
Allograft [13]	Similar to native tissue (150–250 MPa)	Good integration; Risk of immune response	Limited availability; Cost considerations
PCL-GT-GO Graft (Current Study)	240.75 MPa	>80% cell viability; Non-hemolytic; Controlled degradation	No donor site morbidity; Customizable properties

## Data Availability

The original contributions presented in the study are included in the article, further inquiries can be directed to the corresponding authors.
